# An Exploratory Study of Issues in Training Facilitators for Online Training in Advance Care Planning: Mixed Methods Research

**DOI:** 10.3390/nursrep14020075

**Published:** 2024-04-19

**Authors:** Yuko Goto, Hisayuki Miura

**Affiliations:** Department of Home Care and Regional Liaison Promotion, National Center for Geriatrics and Gerontology, Hospital, Obu 474-8511, Aichi, Japan

**Keywords:** facilitator, online skill training, facilitation, mixed methods research, legitimate peripheral participation

## Abstract

Advance care planning (ACP) has attracted increasing research attention in recent years. In Japan, extensive training has been conducted to improve communication through workshops, such as role-playing. In training, including trainee-centered work, the facilitator who assists trainee learning plays an important role. However, only a few studies have focused on the training of facilitators. Therefore, we exploratorily analyzed by the mixed method the differences in the approaches of experienced and inexperienced facilitators during workshops and conducted a study that could contribute to facilitator training in the future. We recorded the comments and attitudes of 12 facilitators who participated in ACP training conducted in 2022. Based on analysis of the obtained data, a distinct difference was confirmed in the progression of trainee-based learning, encouragement to deepen learning among trainees, and trainees’ responses to questions. Thus, this study indicated the importance of having the opportunity for fellow facilitators to learn through facilitation with experienced facilitators and involvement in issue awareness.

## 1. Introduction

With the global aging of the population and diverse values of patients, a need for social change to enhance the variety of treatment and medical options has led to the ongoing development of ACP.

Globally, the definition of ACP continues to develop. However, in recent years, the following description has been often cited: “ACP is an ongoing process that involves investigating and identifying an individual’s values and considering the significance and results of scenarios involving serious illness to define future care, treatment goals, and preferences. It also includes discussing these preferences with family members and medical care providers, appointing a proxy decision-maker, and recording these preferences and options”. Moreover, this has been the landmark definition of ACP [1,2].

Despite lagging considerably behind the situation in the West, efforts have been made in Japan to proactively spread awareness regarding ACP, and the “guidelines on the decision-making process for medical care at the end of life” have been revised. To spread awareness, training has been conducted in various regions throughout Japan [3]. However, only little awareness regarding ACP has been achieved ([4], pp. 32–39, 47), and most specialists showed a low participation rate in ACP training in certain organizations ([4], p. 117). Nurses in acute care hospitals have reported little involvement in ACP because of a lack of education and skill training, time with patients, and barriers arising from workplace culture and communication [5].

Shared decision-making (SDM) has been considered an important skill in implementing ACP [6]. A fact-finding survey of SDM implemented in primary care outpatient services in Japan revealed that the level of SDM among doctors and patients with outpatient nurses was significantly high [7].

Relevant studies have revealed a barrier to multidisciplinary communication, such as communication between doctors and nurses [8,9,10]; these studies have also indicated that multidisciplinary communication training is necessary to overcome such barriers and strengthen collaborations [11]. Therefore, many educational programs have been developed to determine the effect of improving multidisciplinary communication [12]. Certain workshops, such as role-playing and group discussions, are also included [13], and role-playing is considered an effective learning modality in decision-making support [14]. In Japan, programs involving workshops to improve knowledge to practice ACP and other skills [15] and decision-making skills have been developed [6,16]. However, only a few studies have been conducted on facilitators who support trainees and participate in workshops with them. Moreover, facilitators are needed for effective training to improve communication skills [17]. Determining the difference in facilitation based on the level of facilitator experience will help provide adequate education for newly participating facilitators to gain the desired level of experience.

Facilitators have diverse roles, but in our ACP training, we define a facilitator as someone who encourages learners to speak up so that they can learn independently and encourages multiple learners to collaborate so that they can recognize issues and devise resolutions.

Therefore, this study focused on the differences of the skills between experienced and inexperienced facilitators in ACP training using mixed methods.

Then, to improve the quality of ACP training for learning patient-centered ACP practices, we aimed to identify the skills required by facilitators who are responsible for assisting learners in ACP training and examined their education methods.

## 2. Materials and Methods

### 2.1. Study Design

This is an exploratory study using qualitative data in which facilitators who participated in the ACP training recorded their statements and actions during learning support, and quantitative data, such as the time required for the activities.

Upon obtaining basic knowledge as individuals who have completed learning, experienced facilitators acquire knowledge by facilitating the learning process, participating in the “practical community” [18] with new learners and providing support for them. This learning technique of experienced facilitators corresponds to legitimate peripheral participation (LPP) [19,20] in which knowledge is accumulated as a facilitator through involvement with trainees rather than individual learning. 

The study design selected mixed methods using qualitative data to determine the difference between facilitator activities based on the presence of knowledge, experience through LPP, presence of facilitator experience, and quantitative data such as time spent.

Since facilitators are also strongly influenced by the participants they are in charge of facilitating, the qualitative data include many individual elements of facilitators. Therefore, in order to increase the credibility and dependability of the findings obtained from the qualitative data, triangulation of the data was conducted using data from multiple facilitators. In addition, by comparing the quantitative data, i.e., data on the time required for each facilitator’s activities, methodological triangulation was conducted to increase the external validity and objectivity of the findings obtained in this study.

### 2.2. Recruitment and Participation

The present study included data from 12 facilitators who participated as learning supporters of trainees for “the training on SDM competency in ACP”, which was conducted in 2023.

This workshop was not conducted as a study. In this study, the videos recorded during in this workshop were analyzed. All personnel who participated as facilitators in this ACP training have completed this training. Facilitators who have participated in this ACP training as a facilitator once or less are defined as “inexperienced facilitators”, while facilitators who have participated in this training four or more times are defined as “experienced facilitators”.

In total, six experienced facilitators and six individuals without facilitator experience participated in this workshop. In this study, the number of participants with and without facilitator experience was identical; therefore, we included facilitator activity data.

The conditions for facilitator recruitment were as follows: individuals who had previously completed ACP training and those who wished to participate in the training as a facilitator in the role of providing learning support during the workshop.

During the recruitment process, an information session was conducted in which it was stated that facilitator information in ACP training would be used in the evaluation analysis for training assessment.

As a result, these 12 individuals registered and participated as paid volunteers.

Those who decided to participate as facilitators were given a handout containing a facilitators manual and key points. Then, one week to 10 days before the ACP training, all the facilitators were gathered together and, for an hour, were explained the flow of the ACP training, including points to note and important points.

### 2.3. Setting and Data Collection

ACP training was a 210-min program. During this workshop, facilitators only participated in the role-play and not in the lecture conducted by the instructor. Therefore, the duration of their participation was 120 min. The first role-play lasted 45 min, whereas the second role-play lasted 75 min (Table 1).

During the 120-min period, the facilitators played a role in providing learning support during the two role-plays.

Three trainees comprised one team, and each team included one facilitator who provided trainee learning support. In the first and second role-plays, the same facilitator was in charge of the trainees.

The facilitators met 8 days before the workshop to receive a briefing by the instructor on the role of the facilitator, as well as the important points and considerations regarding the workshop, after which they participated in the workshop.

Prior to the workshop, the trainees and facilitators were briefed by the instructor regarding the progression of the role-plays and associated key points.

The researchers and research assistants assessed the involvement of each team. They prepared detailed notes regarding the trainees’ remarks and appearance, as well as the facilitators’ remarks and behaviors. These descriptive data were converted into electronic data by a clerical assistant who had no association with this study. These data were used in the analysis. When converting the descriptive data to electronic data, one row in an Excel file included the remarks, appearance of trainees, behaviors of the facilitators, and inputs in a chronological order.

Before creating these descriptive data, the research assistants were presented with a sample of descriptive data by the researcher and were given a lecture on how to write the data. They then practiced creating descriptive data at least five times using other training videos. The researcher provided feedback on the descriptive data created in the practice sessions to ensure the quality of the descriptive data created.

### 2.4. Data Analysis

We used electronic data from 12 individuals to describe the facilitation of preparations up to the start of the facilitator role-play and the appearance of facilitation for feedback following role-play completion.

Data regarding facilitator expertise, the presence or absence of facilitator experience, sex, and location of the training were compiled into a list, and the facilitator characteristics were noted.

As there was no existing framework to initiate this analysis, we conducted a content analysis using the inductive approach with the role-plays divided into one and two.

First, we recorded the median, mean, minimum, and maximum values for the time required to prepare the role-plays by the six experienced facilitators and six inexperienced facilitators, as well as the time required for feedback following role-play completion.

Next, the facilitators were categorized into experienced and inexperienced individuals. We recorded the facilitators’ behaviors and remarks, as well as the trainees’ remarks and appearances before and after role-plays based on the learning support behavior of trainees who selected a facilitator in advance. Subsequently, we grouped similar behaviors and remarks, which were described in detail, along with the number of individuals.

The qualitative data were manually categorized using the learning aid function of this ACP training. These learning aid functions were explained to the facilitators prior to the training.

In role-play 1 (communication training to converse about the main points of ACP), the facilitation of preparations before the start of the role-play was divided into the following categories and analyzed: (1) **greeting and self-introduction**, (2) **confirmation regarding the way of proceeding**, (3) **encouragement to determine the role-playing part**, and (4) **handling questions and counseling**.

Facilitation of feedback following role-play completion was divided into the following categories and analyzed: (1) encouragement to provide role-play feedback to fellow trainees, (2) explanation of the main points of the feedback, (3) sharing their lessons among fellow trainees through feedback, (4) handling questions and counseling, and (5) time management.

In role-play 2 (communication training of ACP using decision-making support skills), the facilitation of preparations before the start of the role-play was divided into the following categories and analyzed: (1) **confirmation regarding the way of proceeding**, (2) **explanation of the role-playing part**, (3) **decision assistance**, (4) **confirmation of the tools used by the trainee**, and (5) **handling questions and counseling**.

Facilitation of feedback following role-play completion was divided into the following categories and analyzed: (1) confirmation regarding role-play evaluation by each trainee, (2) explanation of the main points of the feedback, (3) sharing their lessons among fellow trainees through feedback, (4) handling questions and counseling, and (5) time management.

Furthermore, integrated content from facilitators with/without experience was presented by category, and the difference was verbalized. To ensure the reliability and validity of our analysis, we repeated the examination until an agreement was reached between two researchers with ≥5 years of experience in ACP education.

### 2.5. Ethical Considerations

This study was conducted in accordance with the Declaration of Helsinki and was approved by the Ethics Committee of the National Institute for Longevity Sciences (ap-proval no. 1585).

This study was conducted using data describing video footage of ACP training. Therefore, no information identifying individual facilitators was collected in this study.

Descriptive data from the training videos were generated by research assistants using the methods used during the training, and the descriptive data were analyzed in this study.

Twelve information sessions were held to recruit facilitator participants.

At these facilitator recruitment briefings, the facilitators were informed that in order to improve this workshop, not only the training results of the participants but also the activity information of the facilitators involved in the training operation would be analyzed and improved, that personally identifiable information would not be used in the research, and that personally identifiable information would not be disclosed. The facilitator clearly explained that those who did not participate as facilitators would not be disadvantaged and that facilitators could decline to participate after registering.

Based on these explanations, participants in the explanatory meeting were asked to understand the purpose of the study, the research methodology for secondary use of workshop information, the voluntary nature of participation, anonymity, and the non-public nature of the data, and to consider participating as a facilitator. Those who wished to participate voluntarily registered to participate in this workshop.

In this study, the Ethics Review Board decided that the voluntary registration of facilitators as participants was regarded as their consent to provide data to this study.

The reason for this was that the facilitators had completed the training course, were familiar with its content, and were healthcare professionals who understood the need for further research.

## 3. Results

The workshop included 36 trainees, and all members completed the training. Overall, 12 facilitators participated in the workshop.

### 3.1. Participants’ Characteristics

Regarding the expertise of the facilitators, of the twelve individuals, four (33%) were nurses, three (25%) were doctors, two (17%) were medical social workers, two (17%) were care managers, and one (8%) was a nutritionist. With regard to sex, six individuals were males (50%) and six were females (50%). The facilitators received previous ACP training at five different training sites (A–E) (Table 2).

### 3.2. Time Required by Each Facilitator for the Role-Play

Regarding the first role-play, of the overall work duration of approximately 35 min, 10 min was assigned to conduct the role-play. Regarding the second role-play, of the overall work duration of approximately 55 min, 15 min was assigned to conduct the role-play.

Each facilitator’s preparation time before the start of the role-plays and the time for feedback after the completion of the role-plays were recorded.

In role-play 1, the preparation time required before the start of the role-play performed by experienced facilitators was a median and mean of 5.5 min, minimum of 3 min, and maximum of 8 min. For inexperienced facilitators, the median was 4.5 min, mean was 5 min, minimum was 3 min, and maximum was 9 min.

Furthermore, for experienced facilitators, the time required for feedback following role-play completion was a median of 16 min, mean of 15.8 min, minimum of 14 min, and maximum of 17 min. For inexperienced facilitators, the median was 16.5 min, mean was 15.7 min, minimum was 13 min, and maximum was 20 min (Table 3).

In role-play 2, the preparation time required before the start of the role-play by experienced facilitators was a median and mean of 6.5 min, minimum of 5 min, and maximum of 8 min. For inexperienced facilitators, the median was 8.5 min, mean was 7.8 min, minimum was 1 min, and maximum was 12 min. Furthermore, for experienced facilitators, the time required for feedback following role-play completion was a median of 27 min, mean of 28.2 min, minimum of 25 min, and maximum of 32 min. For inexperienced facilitators, the median was 27.5 min, mean was 27.3 min, minimum was 24.0 min, and maximum was 32.0 min (Table 4).

### 3.3. Classification of the Remarks and Behaviors of Facilitators during Role-Plays

In this study, facilitators who have participated in this ACP training as a facilitator once or less are defined as “inexperienced facilitators”. We defined facilitators who had participated in this ACP training four or more times as “experienced facilitators”.

#### 3.3.1. Role-Play 1: Communication Training to Converse about the Main Points of ACP

In role-play 1, 163 and 146 sentences were obtained from experienced and inexperienced facilitators, respectively.

Facilitation of preparations before the start of role-plays was divided into the following four categories and analyzed.

Regarding **greetings and self-introduction**, the process was performed by all facilitators, including those with and without experience. The trainees were then encouraged to provide a self-introduction, and the difference between experienced and inexperienced facilitators was confirmed.

Regarding **confirmation of the proceeding method**, experienced facilitators determined the role-play part while listening to the opinions of all trainees, explained the method and time schedule, and ensured that all trainees understood.

One of the six inexperienced facilitators provided a briefing on the proceeding method that differed from the instructor’s explanation, which was indicated by the trainees.

Regarding **encouragement to determine the role-play part**, all experienced facilitators encouraged role determination while recording the trainees’ opinions and reactions. Two of the six inexperienced facilitators determined the use of role-play and provided instructions to the trainees. Furthermore, one of the six inexperienced facilitators required considerable time because they could not progress in role determination.

Regarding **handling questions and consultations**, four of the six experienced facilitators received inquiries from trainees and responded to them. Five of the six inexperienced facilitators received point-out questions from the trainees, and one of the five provided an incorrect answer. In contrast, two of the five inexperienced facilitators did not respond to the questions and instead shared their personal views and opinions.

**Facilitation of feedback following role-play completion** was divided into the following five categories and analyzed.

Regarding **encouragement to provide role-play feedback to fellow trainees**, all experienced facilitators encouraged trainees to comment on their impressions following role-play completion and then asked questions about the specifics of the remarks.

All inexperienced facilitators encouraged the trainees to comment on their impressions following role-play completion. After obtaining remarks from the trainees, five of the six inexperienced facilitators provided their personal impressions. Three of the six inexperienced facilitators asked questions to the trainees who were interested in the facilitator’s personal experience and the facilitators themselves.

Regarding the **explanation of the main points of feedback**, five of the six experienced facilitators explained the main points of the feedback and encouraged the trainees to provide remarks. Moreover, one of the six inexperienced facilitators explained the main points of the feedback and encouraged the trainees to provide remarks.

Regarding **sharing their lessons among fellow trainees through feedback**, all experienced facilitators obtained information from their trainees about the activity settings of all trainees and promoted discussions on the use of role-play in learning.

Furthermore, while responding to questions, they encouraged fellow trainees to share their views on the differences between trainees.

Two of the six inexperienced facilitators obtained information from the trainees about their activity settings and promoted discussions on the use of role-play in learning. Five of the six inexperienced facilitators expressed their own impressions, during which the trainees listened to the one-sided remarks on the facilitators.

Regarding **handling questions and consultations**, three of the six experienced facilitators received spontaneous questions from the trainees. After the facilitators expressed their personal opinions, they encouraged other trainees to express their respective opinions.

Two of the six inexperienced facilitators received spontaneous questions from the trainees. Among them, one individual did not respond but immediately encouraged other trainees to express their opinions, whereas the other individual did not answer the trainees’ questions.

Five of the six experienced facilitators concluded the **time management** session with a discussion regarding the specified time. All six inexperienced facilitators concluded with a discussion about the specified time.

#### 3.3.2. Role-Play 2: Communication Training of ACP Using Decision-Making Support Skills

In role-play 2, the experienced facilitators extracted 180 sentences, and the inexperienced facilitators extracted 197 sentences.

In role-play 2, **facilitation of preparation before the start of the role-play** was divided into four categories and analyzed.

Regarding **confirmation of the proceeding method**, all experienced facilitators explained and confirmed the proceeding method. Five of the six inexperienced facilitators explained the procedure.

Regarding **assistance for the explanation and determination of the role-playing part**, all experienced facilitators answered the trainees’ questions and supported their roles in the decision-making process. Four of the six inexperienced facilitators encouraged the trainees to make role decisions. Moreover, while answering the questions several times, they supported the trainees in their role decision-making. Two of the six inexperienced facilitators determined their roles and instructed the trainees.

**Confirmation of the tools used by the trainees** is the process of confirming the scenario used in the role-play and the worksheet. After the role was determined, all experienced facilitators performed both confirmation processes. Five of the six inexperienced facilitators conducted both confirmation processes following role determination. One of the six inexperienced facilitators provided an incorrect explanation, which was corrected by the instructor.

Regarding **handling questions and consultations**, four of the six experienced facilitators received spontaneous questions from the trainees, which were answered correctly. Moreover, they discussed the trainees’ difficult expressions, provided further explanations after watching them carefully read the text at hand, and discussed the points explaining the anxiety of the trainees. Five of the six inexperienced facilitators received random questions from the trainees and responded; however, they answered while checking the text and spoke in a very timid voice.

Facilitation of feedback after role-play completion was divided into five categories and analyzed.

With regard to **confirmation regarding the role-play evaluation by each trainee**, all facilitators, including those with and without experience, shared the role-play results using a worksheet per person. Furthermore, five of the six experienced facilitators ensured that the time for providing feedback, including that for fellow trainees to ask each other questions, was at least 10 min. Three of the six inexperienced facilitators ensured that the duration was at least 10 min and that the time for providing feedback was set aside, such as that for fellow trainees to ask each other questions.

With regard to the **explanation of the main points of feedback**, four of the six experienced facilitators encouraged providing feedback after explaining the main points of feedback, whereas three of the six inexperienced facilitators encouraged providing feedback after explaining the main points of the feedback.

Regarding **sharing their lessons among fellow trainees through feedback**, all experienced facilitators confirmed the actual activity status of the trainees and proceeded to discuss on how to use the present learning. All inexperienced facilitators attempted to confirm the actual activity status of the trainees; however, all six individuals confirmed that the conversation for feedback in accordance with the training purpose did not continue for reasons such as the trainee narrations differed from the feedback and the trainees’ narrations ended easily, resulting in continued silence.

Regarding **handling questions and consultations**, three of the six experienced facilitators received independent questions from the trainees and responded appropriately. Furthermore, they encouraged queries to be directed at the instructor. Five of the six inexperienced facilitators received questions from the trainees. One of these five individuals received repeated questions because they could not understand the facilitator’s answer. Two of these five individuals could not respond and provided the facilitator’s impressions. Moreover, one of the five individuals interrupted the trainees’ questions and discussed their personal experiences.

With regard to **time management**, all facilitators, including those with and without experience, completed the work within the given time. The remaining time was communicated orally on a regular basis, resulting in time management.

## 4. Discussion

In terms of the ACP training in this study, we investigated the difference between experienced and inexperienced facilitators. The difference in facilitation based on the presence or absence of facilitator experience articulated and depicted the time difference in how work progressed and the phenomenon of learning support for trainees.

### 4.1. Differences in Work Time Management

In role-play 1, the median and mean preparation times until the start of the role-play differed by less than 1 min for experienced and inexperienced facilitators. As the minimum value was the same at 3.0 min and the maximum value differed by 1 min, no major difference was observed between the experienced and inexperienced facilitators.

The feedback time following the completion of the role-play in role-play 1 differed by less than 1 min in terms of the median, mean, and minimum times. However, the maximum duration for experienced facilitators was 3 min less than that for inexperienced facilitators. Therefore, in the time management for feedback, we observed more variation among inexperienced facilitators.

In role-play 2, compared with inexperienced facilitators, the median preparation time before the start of the role-play was 2 min less, whereas the mean preparation time was 1.2 min less for experienced facilitators. The difference between the minimum and maximum times was 3 min for experienced facilitators but 7 min for inexperienced facilitators, and a greater variation in time management was observed among inexperienced facilitators.

The feedback time following the completion of the role-play in role-play 2 differed by less than 1 min between the experienced and inexperienced facilitators in terms of the median and mean times. The minimum time had a difference of less than 1 min, and the maximum time remained the same.

Based on the time differences between role-plays 1 and 2, the time for role-play implementation was uniform for experienced facilitators and was appropriately managed. The inexperienced facilitators showed a major variation in the role-play preparation and feedback times, and such time variation was adjusted by the trainee’s role-play time.

Previous research has shown that self-performance ratings are higher when people are aware that they can manage their time [21]. The facilitator’s ability to manage time appropriately is expected to increase the facilitator’s confidence and further enhance the facilitator’s performance in supportive learning, which is beneficial to the trainees.

The results revealed several differences between experienced and inexperienced facilitators. Especially, clear differences were observed in the performance of learning support for participants. Therefore, it is necessary to provide appropriate advice to each facilitator to improve their skills and provide relevant support.

### 4.2. Differences in the Involvement with Trainees

With regard to the two types of role-plays, we further discuss the **facilitation of preparation before starting the role-plays**.

With regard to **confirmation regarding the proceeding method**, the experienced facilitators explained the proceeding method and confirmed it with the trainees, whereas the inexperienced facilitators provided incorrect explanations of the proceeding method, and some did not confirm the method with the trainees, which caused a difference in the accuracy of the explanation of the proceeding method. To standardize the quality of training, such differences have a large impact on trainees. Thus, facilitator training requires a thorough explanation of the proceeding method.

Regarding **encouragement to determine the role-play part** and **explanation of role-playing and decision assistance**, all experienced facilitators provided support to encourage trainee-centered role decisions. Although the inexperienced facilitators determined the trainees’ roles and confirmed the designated cases, the trainee-centered role determination was not thorough. Therefore, the underlying reason that led the facilitator to determine the role must be confirmed and applied in facilitator training.

Regarding the **confirmation of the tools used by the trainees** conducted in role-play 2, the inexperienced facilitators provided incorrect explanations of the tools, indicating that the facilitators did not fully understand the method of tool usage. This point should be thoroughly discussed in facilitator training.

A significant difference was observed in **handling questions and consultations**. After experienced facilitators described their thoughts and knowledge, other trainees conducted discussions in which they could express their thoughts. Furthermore, based on the trainees’ expressions and behaviors, questions were raised and trainee difficulties were deduced.

Inexperienced facilitators expressed their personal impressions only in response to the questions, provided incorrect responses, and gave brief answers while searching through texts. By observing the state of the individual who was asking the question, it was inferred that they were troubled and did not appear to encourage further questions. This indicates a difference in the trainee’s ability to manage and accumulate knowledge while observing his/her appearance, as well as a difference in the facilitator’s ability to deepen discussions through experience.

Therefore, opportunities must be created for inexperienced facilitators to learn through facilitation with experienced individuals.

Next, we discuss the facilitation of feedback following role-play completion.

Regarding the **explanation of the main points of feedback**, the experienced and inexperienced facilitators did not perform such tasks thoroughly. The explanation was provided to the trainees by the instructor in advance. However, by explaining the main points of feedback thoroughly to the trainees again by facilitators, it was expected that the trainees will be able to provide comments easily. The **explanation of the main points of feedback** should be performed thoroughly during facilitator training.

We confirmed that all facilitators, both experienced and inexperienced, were engaged in **encouragement to provide role-play feedback to fellow trainees** and **confirmation regarding the role-play evaluation by each trainee**. With regard to differences between experienced and inexperienced facilitators, experienced facilitators could turn to other trainees after collecting information from trainees. In contrast, inexperienced facilitators described their personal impressions, which led to the discussion of topics of their own interest. This revealed that inexperienced participants were more likely to engage in facilitator-centered facilitation rather than student-centered facilitation, and this finding should be incorporated into facilitator training.

**Sharing their lessons among fellow trainees through feedback** was adopted by all facilitators, including experienced and inexperienced facilitators. However, for inexperienced facilitators, it served as a forum for sharing facilitator experiences, and we confirmed a phenomenon in which the inexperienced facilitators could not effectively facilitate discussions with trainees and did not deepen trainee remarks. Conversational training that promotes trainee awareness through a two-way dialog is essential. Therefore, inexperienced facilitators require opportunities to learn while experiencing effective facilitation with experienced individuals.

Regarding **handling questions and consultations**, a clear difference was observed between experienced and inexperienced facilitators. Inexperienced facilitators discussed a different topic without answering the trainees’ questions, and we confirmed a phenomenon in which they provided answers that the trainees could not understand. When they could not personally answer a question, the experienced facilitators encouraged that the questions should be addressed to the instructor and handled situations by incorporating such questions into the trainees’ discussions. Answering questions appropriately leads to trainee learning, as well as facilitator learning. Therefore, with regard to answering trainee questions, it is important to respond thoroughly in facilitator training.

Although the role of facilitators in supporting trainees’ learning is crucial, it has been emphasized that only support for improving their skills is insufficient [22]. Previous research has highlighted the importance of iterative learning processes in improving facilitator skills [23]. By repeating a workshop in which an inexperienced facilitator takes on the role alone and a workshop together with an experienced facilitator, the overall skills of the facilitator can be expected to improve.

### 4.3. Training to Eliminate the Difference in Facilitation between Experienced and Inexperienced Individuals

In this study, we determined the differences according to the experience of facilitators who support trainee learning assistance.

As the facilitators’ learning support abilities play an important role in simulation education [17], it is important to standardize the facilitators’ learning support abilities. Facilitators must appropriately understand the needs of the learners and respond flexibly [24].

Factors that confirmed a major difference between experienced and inexperienced facilitators based on the presence or absence of facilitator experience included the trainee-centered work proceeding method and facilitator-centered work proceeding method. ACP is based on patients’ values, and it was suggested that performing training through a facilitator-centered educational approach is inappropriate. Thus, in relation to the trainee-centered measures, training for facilitators should be comprehensive.

Communication that elicits trainee remarks provides conceivable opportunities to seek comments from other trainees. Furthermore, when handling questions from trainees, we confirmed that inexperienced facilitators did not answer at all or answered inappropriately, which resulted in improper trainee learning.

Trainee queries were often difficult to address, and facilitators learn such conversational skills through practice. This study did not determine the type of process that improves the facilitators’ abilities. Therefore, further research should be conducted in the future to help develop a training program to support facilitator abilities.

There was no significant difference in the time required for the workshop between the experienced and inexperienced facilitators, and because of the facilitator-centered facilitation, the inexperienced participants concentrated on following the prescribed schedule. This may be partly due to the fact that the inexperienced facilitators did not have time to objectively capture, interpret, and learn from the trainee’s information. To provide more time, it may be effective to coordinate the placement of facilitators who can interact with the learners along with experienced facilitators.

### 4.4. For Future Implications and Limitations of This Study

This study has several limitations. The data used in this study were secondary descriptive data generated and analyzed from video data of ACP trainings that were not intended for research. Therefore, the generalizability of the results of this study is severely limited by the fact that the analysis did not include the strict educational background of the facilitators or the influence of facilitator experience outside of this ACP training. This study was conducted by the researcher who developed this ACP training program and analyzed the activities of 12 facilitators, which may have caused some biases in the results. The researchers who developed this ACP training program know the program better than anyone else. The amount of information obtained is noticeably different from the facilitators’ experience, and it is possible that subtle differences and issues among facilitators may not have been fully extracted.

As an implication for the future, our study is one of the few that explored the skills required by facilitators in ACP training to personnel development. We believe that it is crucial to examine methods for learner-centered facilitation in training for ACP practice, which needs to be conducted in a patient-centered setting. In the future, it will be necessary to design a facilitator training program as a study and accumulate evidence with higher generalizability.

## 5. Conclusions

In the present study focusing on facilitators who support trainee learning in ACP training, a difference in facilitation based on the level of experience of the facilitator was observed. Factors that confirmed a major difference between experienced and inexperienced facilitators based on the presence or absence of facilitator experience included the trainee-centered work proceeding method and facilitator-centered work proceeding method.

The results suggest the need for improved educational interventions for facilitators who provide facilitator-centered learning in patient-centered ACP practices.

## Figures and Tables

**Table 1:** Role-play program with facilitators participating as learning assistants.

Role-Play Type	Time (min)	Details	Learning Assistance Behavior for Trainees Who Were Designated a Facilitator in Advance (Analysis Category)
First role-play Communication training to discuss the main points of ACP			
Setting the patient role: an elderly patient with vascular dementia who was living alone had considerably reduced ADL and auditory acuity	10	The instructor will explain how the role-play will proceed	
Expert role: setting the place where a discussion regarding the treatment and care of these patients will be conducted Observer role: observation and feedback will be provided without the participation of a trainee in the role-play	10	Preparations up to the start of the role-play	**Greeting and self-introduction** **Confirmation regarding the way of proceeding** **Encouragement to determine the role-playing part** **Handling questions and counseling**
	10	Role-play implementation	Observation of the appearance of the trainee Time management
	15	Feedback following the completion of the role-play	**Encouragement to provide role-play feedback to fellow trainees** **Explanation regarding the main points of the feedback** **Sharing their lessons among fellow trainees through feedback** **Handling questions and counseling** **Time management**
Rest break	10		
Second role-play Communication training for ACP using decision-making support skills			
Setting the patient role: an elderly patient with symptoms of cognitive dysfunction and pneumonia who did not seek a doctor The patient lived with one son (a recluse) The patient’s brother lived in the neighborhood, but they are not close The patient had many good friends in the neighborhood	20	The instructor will explain how the role-play will proceed	
Setting the story: the patient met with a traffic accident, and this was the first time that they sought a specialist Setting: first meeting The trainee will participate in the role-play by playing the patient, specialist, and third-party roles (e.g., family and friends)	10	Preparations up to the start of the role-play	**Confirmation regarding the way of proceeding** **Explanation regarding the role-playing part and decision assistance** **Confirmation regarding the tools used by the trainee** **Handling questions and counseling**
	15	Role-play implementation	Observation of the appearance of the trainee Time management
	25	Feedback following the completion of the role-play	**Confirmation regarding role-play evaluation by each trainee** **Explanation of the main points of the feedback** **Sharing their lessons among fellow trainees through feedback** **Handling questions and counseling** **Time management**

The bold text indicates categories for qualitative analysis.

**Table 2:** Facilitator characteristics (N = 12).

	Expertise	Experience as a Facilitator (with/without)	Sex	ACP Training Venue
1	Nurse	With	F	C
2	Nurse	With	F	C
3	Nurse	Without	F	A
4	Nurse	Without	F	E
5	Doctor	With	M	B
6	Doctor	With	M	A
7	Doctor	Without	M	D
8	Medical social worker	With	M	B
9	Medical social worker	Without	M	D
10	Care manager	With	F	B
11	Care manager	Without	M	D
12	Nutritionist	Without	F	A

**Table 3:** Preparation time required before the start of the role-play and the time required for feedback following the completion of role-play 1 (N = 12).

		Experienced (min)	Inexperienced (min)
Time required for preparation up to the start of the role-play	Median	5.5	4.5
	Mean	5.5	5.0
	Minimum	3.0	3.0
	Maximum	8.0	9.0
Time required for feedback following role-play completion	Median	16.0	16.5
	Mean	15.8	15.7
	Minimum	14.0	13.0
	Maximum	17.0	20.0

**Table 4:** Preparation time required before the start of the role-play and the time required for feedback following the completion of role-play 2 (N = 12).

		Experienced (min)	Inexperienced (min)
Time required for preparation before the start of the role-play	Median	6.5	8.5
	Mean	6.5	7.7
	Minimum	5.0	1.0
	Maximum	8.0	12.0
Time required for feedback following role-play completion	Median	27.0	27.5
	Mean	28.2	27.3
	Minimum	25.0	24.0
	Maximum	32.0	32.0

## Data Availability

The data presented in this study are available upon request from the corresponding author.

## Public Involvement Statement

No public involvement in any aspect of this research.

## Guidelines and Standards Statement

This manuscript was drafted against a checklist to improve reporting of group-based behavior-change interventions [25] for this mixed methods research.

## References

[1] Monnet F., Pivodic L., Dupont C., Dröes R.M., Van den Block L. (2022). Information on advance care planning on websites of dementia associations in Europe: A content analysis. Aging Ment Health.

[2] Sudore R.L., Lum H.D., You J.J., Hanson L.C., Meier D.E., Pantilat S.Z., Matlock D.D., Rietjens J.A.C., Korfage I.J., Ritchie C.S. (2017). Defining advance care planning for adults: A consensus definition from a multidisciplinary Delphi panel. J. Pain Symptom. Manag..

[3] Ministry of Health, Labour and Welfare Guidelines on the Decision-Making Process for Medical Care at the End of Life. https://www.mhlw.go.jp/file/06-Seisakujouhou-10800000-Iseikyoku/0000197721.pdf.

[4] Study Meeting on How Awareness Should Be Spread Regarding Medical Care in the Final Stage of Life Report of the Opinion Poll Regarding Medical Care in the Final Stage of Life. https://www.mhlw.go.jp/toukei/list/dl/saisyuiryo_a_h29.pdf.

[5] McCourt R., James Power J., Glackin M. (2013). General nurses’ experiences of end-of-life care in the acute hospital setting: A literature review. Int. J. Palliat. Nurs..

[6] Goto Y., Miura H., Yamaguchi Y., Onishi J. (2022). Evaluation of an advance care planning training program for practice professionals in Japan incorporating shared decision making skills training: A prospective study of a curricular intervention. BMC Palliat. Care.

[7] Goto Y., Miura H., Son D., Scholl I., Kriston L., Härter M., Sato K., Kusaba T., Arai H. (2021). Association between physicians’ and patients’ perspectives of shared decision making in primary care settings in Japan: The impact of environmental factors. PLoS ONE.

[8] Weber S., Courtney K.L., Benham-Hutchins M. (2009). Decision support in multi-professional communication. J. Med. Syst..

[9] Robinson M., Cottrell D. (2005). Health professionals in multi-disciplinary and multi-agency teams: Changing professional practice. J. Interprof. Care.

[10] Walker A., Breitsameter C. (2022). Medical decision-making in hospices from the viewpoint of physicians: Results from two qualitative studies. BMC Palliat. Care.

[11] Lancaster G., Kolakowsky-Hayner S., Kovacich J., Greer-Williams N. (2015). Interdisciplinary communication and collaboration among physicians, nurses, and unlicensed assistive personnel. J. Nurs. Scholarsh..

[12] Chung H.O., Oczkowski S.J., Hanvey L., Mbuagbaw L., You J.J. (2016). Educational interventions to train healthcare professionals in end-of-life communication: A systematic review and meta-analysis. BMC Med. Educ..

[13] Chan C.W.H., Ng N.H.Y., Chan H.Y.L., Wong M.M.H., Chow K.M. (2019). A systematic review of the effects of advance care planning facilitators training programs. BMC Health Serv. Res..

[14] Kanzaria H.K., Chen E.H. (2020). Shared decision making for the emergency provider: Engaging patients when seconds count. MedEdportal.

[15] Okada H., Morita T., Kiuchi T., Okuhara T., Kizawa Y. (2021). Health care providers’ knowledge, confidence, difficulties, and practices after completing a communication skills training program for advance care planning discussion in Japan. Ann. Palliat. Med..

[16] Goto Y., Miura H. (2023). Evaluation of an advanced care planning training program incorporating online skills in shared decision making: A preintervention and postintervention comparative study. Healthcare.

[17] Heffernan R., Brumpton K., Randles D., Pinidiyapathirage J. (2021). Acceptability, technological feasibility and educational value of remotely facilitated simulation based training: A scoping review. Med. Educ. Online.

[18] Lave J., Wenge E. (2002). Legitimate peripheral participation in communities of practice. Distributed Learning.

[19] Sutherland L.M., Scanlon L.A., Sperring A. (2005). New directions in preparing professionals: Examining issues in engaging students in communities of practice through a school–university partnership. Teach. Teach. Educ..

[20] Fuller A., Hodkinson H., Hodkinson P., Unwin L. (2005). Learning as peripheral participation in communities of practice: A reassessment of key concepts in workplace learning. Br. Educ. Res. J..

[21] Macan T.H., Shahani C., Dipboye R.L., Phillips A.P. (1990). College students’ time management: Correlations with academic performance and stress. J. Educ. Psychol..

[22] Delisle V.C., Gumuchian S.T., Kloda L.A., Boruff J., El-Baalbaki G., Körner A., Malcarne V.L., Thombs B.D., Scleroderma Support Group Project Advisory Team (2016). Effect of support group peer facilitator training programmes on peer facilitator and support group member outcomes: A systematic review. BMJ Open.

[23] Chou C.L., Hirschmann K., Fortin A.H., Lichstein P.R. (2014). The impact of a faculty learning community on professional and personal development: The facilitator training program of the American Academy on Communication in Healthcare. Acad. Med..

[24] Solli H., Haukedal T.A., Husebø S.I.E., Reierson I.Å. (2022). Alternating between active and passive facilitator roles in simulated scenarios: A qualitative study of nursing students’ perceptions. Adv. Simul..

[25] Borek A.J., Abraham C., Smith J.R., Greaves C.J., Tarrant M. (2015). A checklist to improve reporting of group-based behaviour-change interventions. BMC Public Health.

